# Antiphospholipid Syndrome and Patent Foramen Ovale: A Case Report and Literature Review

**DOI:** 10.7759/cureus.61539

**Published:** 2024-06-02

**Authors:** Maria Rocha, Francisca A Correia, Maria Inês Matos, Sergio Madureira, Ana Neves

**Affiliations:** 1 Internal Medicine, Centro Hospitalar e Universitário de São João, Porto, PRT

**Keywords:** patent foramen ovale, catastrophic antiphospholipid syndrome, antiphospholipid antibodies, autoimmune disease, antiphospholipid syndrome

## Abstract

Antiphospholipid syndrome (APS) is a systemic autoimmune disease characterized by arterial, venous, or microvascular thrombosis, pregnancy morbidity, or non-thrombotic manifestations in patients with persistent antiphospholipid antibodies (aPL). Catastrophic APS is a rare and severe form of APS that is defined by the presence of multiple vascular occlusive events. When a patent foramen ovale (PFO) is present, paradoxical embolization can occur, simultaneously leading to arterial and venous thrombosis. We present a complex clinical case of a patient who presented with multiple arterial and venous thrombotic events with positive aPL. The suspicion of catastrophic APS was removed when a PFO was found in a transesophageal echocardiogram, justifying paradoxical embolization. This emphasizes the importance of searching for PFO in patients with APS presenting with simultaneous venous and arterial thrombosis for management and prognosis purposes.

## Introduction

Antiphospholipid syndrome (APS) is a systemic autoimmune disease characterized by arterial, venous, or microvascular thrombosis, pregnancy morbidity, or non-thrombotic manifestations in patients with persistent antiphospholipid antibodies (aPL) [1]. The classification of APS is based on the classification criteria published in 2023 by the American College of Rheumatology and the European Alliance of Associations for Rheumatology, which classifies a patient as having APS when they have at least one clinical criteria from arterial or venous macrovascular thrombosis, microvascular thrombosis, obstetric mortality, cardiac valve abnormalities, or thrombocytopenia plus aPL test within three years from the clinical criteria plus a score of ≥3 in clinical criteria plus a score of ≥3 in laboratory criteria. Catastrophic APS (CAPS) is a rare and severe form of APS that is defined by the presence of (1) multiple vascular occlusive events (three or more), (2) development of manifestations in less than one week, (3) confirmation by histopathology of occlusion in ≥1 organ or tissue, and (4) persistent presence of aPL over 12 weeks [2]. When a patent foramen ovale (PFO) is present, paradoxical embolization can occur, leading to arterial and venous thrombosis simultaneously [3]. The association between APS and PFO has been reported [4-5], and it can be misdiagnosed as CAPS when present. The management of those patients is challenging, both in the acute phase and later in the decision for closure intervention. We report a case of an APS presenting with multiple arterial and venous thrombotic manifestations because of the concomitant presence of a PFO, and we did a review of how to manage those patients.

## Case presentation

**Figure 1:**
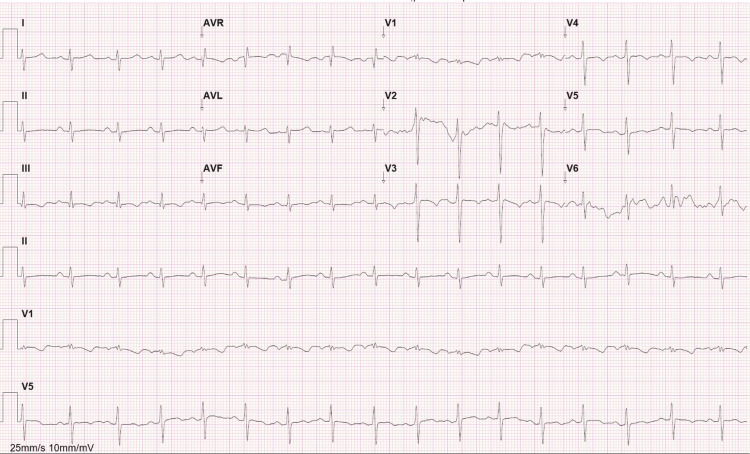
Electrocardiogram in the emergency room

A 33-year-old male patient was admitted to the emergency room due to progressive dyspnea for two months associated with fever and myalgias in the last 24 hours. While waiting for medical observation, he developed right-eye amaurosis and diplopia. On physical examination, he had low blood pressure, tachycardia, and a temperature of 37.7 ºC, and he was tachypneic with a peripheral saturation of 89% while breathing room air. On a neurologic exam, he could count fingers but had impaired visual acuity with the right eye, in addition to right abducens paresis and a minimal afferent pupillary defect. Pulmonary auscultation was normal, and he had jugular vein distension and capillary reperfusion of four seconds. The lower limbs were cold, and peripheral pulses were symmetrical but filiform. Arterial blood gas analysis showed severe hypoxemic respiratory failure and slightly high lactic acid, and the blood analysis evidenced normal platelets, normal coagulation tests, slightly increased C-reactive protein, and normal myocardial necrosis markers (Table 1). The electrocardiogram showed sinus tachycardia and the typical sign S1Q3T3 (Figure 1).

**Table 1:** Blood tests in the emergency room

Blood tests	Results	Reference range
Hemoglobin (g/dL)	14.3	13-17
White blood cells (x109/L)	10.7	4-11
Platelets (x109/L)	189	150-450
Urea (mg/dL)	25	5-30
Creatinine (mg/dL)	0.73	0.7-1.3
C-reactive protein (mg/L)	32	<5
Troponin I (ng/L)	<30	-
Brain natriuretic peptide (pg/mL)	<30	-
Activated partial thromboplastin time (seconds)	34	21-35
Prothrombin time (seconds)	12	11-13.5

**Figure 2:**
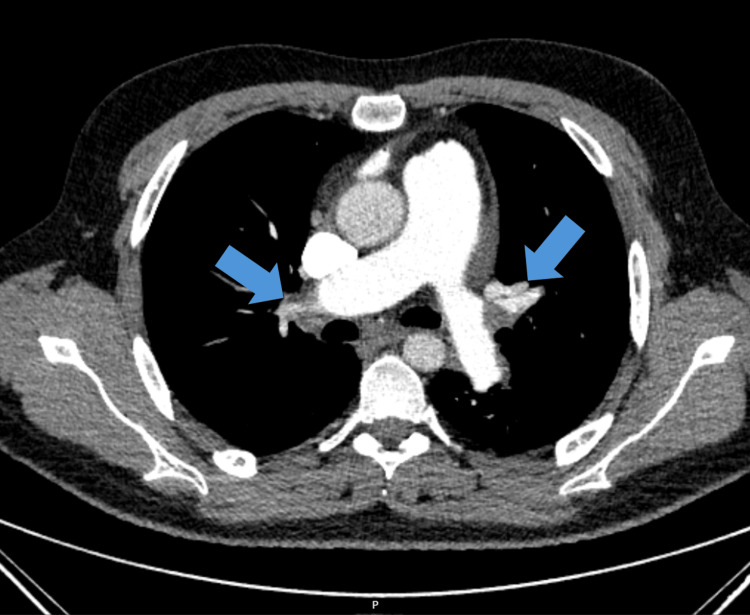
Thoracic CTA in the emergency room showing the filling defects (blue arrows) in the right pulmonary artery and a branch of the left pulmonary artery CTA: computed tomography angiography

Regarding the high probability of pulmonary embolism, the patient was submitted to a thoracic computed tomography angiography (CTA), and for the neurologic deficits, he was also submitted to a brain CTA. Meanwhile, non-invasive mechanical ventilation was started. The thoracic CTA evidenced bilateral filling defects in branches of the pulmonary arteries (Figure 2).

**Figure 3:**
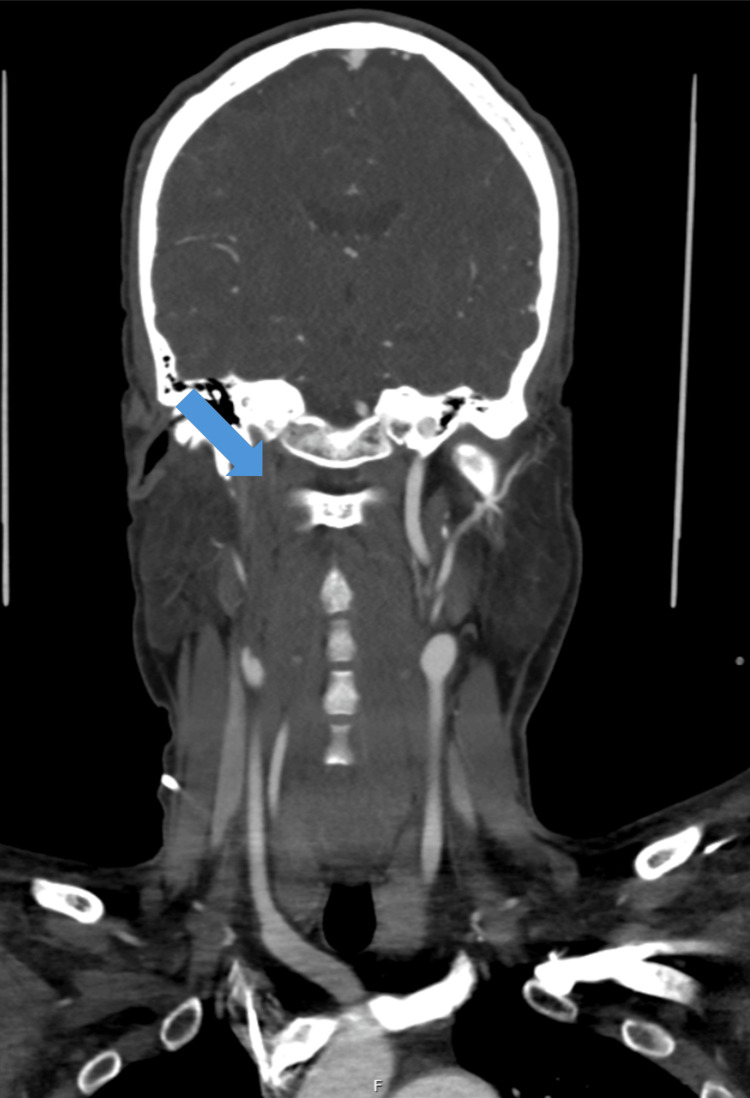
Brain CTA in the emergency room showing the total obliteration (blue arrow) of the right internal carotid artery CTA: computed tomography angiography

The brain CTA showed right internal carotid artery occlusion (Figure 3). We performed a transcranial and neck vessel Doppler ultrasound that showed right-resistance flow in the right internal carotid artery and the right ophthalmic artery, suggesting thrombosis. To stratify the risk, a transthoracic echocardiogram was performed that showed right ventricular dysfunction. Lower limb Doppler ultrasound was also performed, which revealed arterial and venous popliteal thrombosis.

He had a good response to the non-invasive mechanical ventilation and remained stable. Anticoagulation was started with unfractionated heparin, and he was admitted to the ICU for multiple arterial and venous thrombosis, namely intermediate-high-risk pulmonary embolism, popliteal arterial and venous thrombosis, right internal carotid artery, and right ophthalmic artery thrombosis. The etiological study showed triple-positive aPL (Table 2), the platelets remained normal, and there were no signs of microvascular embolism.

**Table 2:** Autoimmunity blood tests

Autoimmunity bloodtests	Results	Normal range
Lupus anticoagulant	Positive	-
IgG anticardiolipin antibodies (MPL/mL)	71	<40
IgM anticardiolipin antibodies	12	<40
IgG beta2-glycoprotein 1 antibodies (U/mL)	43	<40
IgG beta2-glycoprotein 1 antibodies	10	<40
Antinuclear antibodies	Negative	-
Anti-dsDNA antibodies	Negative	-
Antineutrophil cytoplasmatic antibodies	Negative	-

Despite that, given the severity of the clinical condition, a high dose of corticosteroids was started on the suspicion of CAPS. The hypoxemic respiratory failure improved just partially after two weeks of anticoagulation, with the thoracic CTA showing signs of persistent subacute pulmonary embolism. A right heart catheterization was performed, displaying severe pulmonary precapillary hypertension (Table 3). During this procedure, thrombotic material was aspirated from branches of bilateral pulmonary arteries, and thrombolysis with intra-arterial alteplase was performed with partial reperfusion.

**Table 3:** Right heart catheterization hemodynamic measures Severe pre-capilar group 4 pulmonary hypertension

Hemodynamic measures	Results
Mean pulmonary heart pressure (mmHg)	53
Mean pulmonary wedge pressure (mmHg)	<15
Pulmonary vascular resistance (WU)	8.3
Cardiac index (lpm/m2)	2.3

The patient was also subjected to a transesophageal echocardiogram that identified a right-left shunt compatible with a PFO (Figure 3). The patient was discharged from UCI to the internal medicine ward with mild hypoxemic respiratory failure requiring 1 liter of oxygen per minute but with severe intolerance to exercise. Treatment with riociguat and ambrisentan was started with tolerance, and anticoagulation was switched to warfarin. The corticosteroids were stopped right after the evidence of the PFO, with no complications. He was discharged from the internal medicine ward to a rehabilitation center. The aPL was repeated after 12 weeks, confirming the triple positivity. It was decided against the closure of PFO and to maintain antithrombotic therapy.

**Figure 4:**
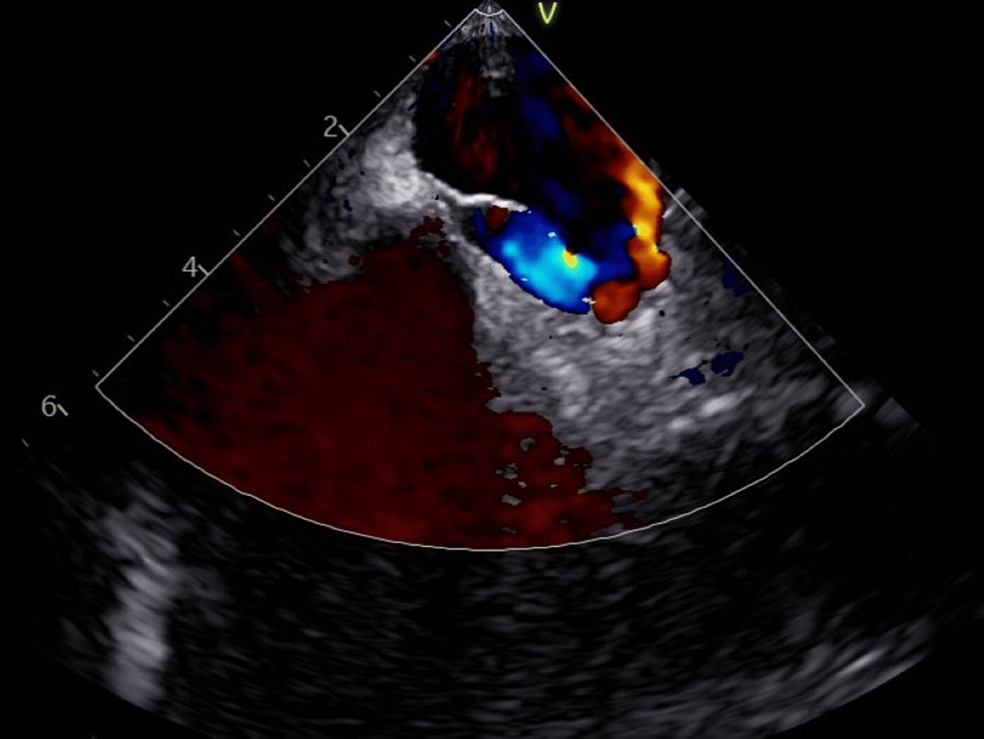
PFO with right-left shunt in transesophageal echocardiogram with color Doppler PFO: patent foramen ovale

## Discussion

APS is a systemic acquired autoimmune thrombophilia characterized by arterial, venous, or microvascular thrombosis, pregnancy morbidity, or non-thrombotic manifestations in patients with persistent presence of aPL [1]. It has been suggested that PFO is more prevalent among patients with thrombophilia like APS, and aPL was reported to be present more frequently in patients proposed for PFO occlusion [4-5]. The management of those patients is challenging, starting with a diagnosis that can be difficult to distinguish from CAPS. CAPS is a rare and life-threatening condition that is characterized by the presence of aPL in a patient with multiple thromboses. However, this condition is not only caused by direct occlusion of the vessel; it’s also a cytokine storm [6]. CAPS most frequently presents with renal failure with hematuria and proteinuria and variable degrees of hypertension caused by microthrombosis [6-7]. Lungs are the second most common affected organ, and stroke is present in almost half of the patients [7]. We presented a clinical case of a young male patient who presented with multiple thrombosis and positive aPL and whose first suspicion was CAPS. However, the evidence of a PFO changed our belief in this diagnosis once our patient did not have any microthrombotic events, thrombocytopenia, or anemia. The evidence of a PFO justified the paradoxical embolism, and the absence of complications after stopping steroid therapy also supports this hypothesis. This emphasizes the importance of searching for PFO in patients with APS presenting with simultaneous venous and arterial thrombosis for management and prognosis purposes. Regarding the closure of the PFO, the international guidelines recommend against closure in persons with thrombophilia, in those for whom antithrombotic therapy is the standard of care [8]. This recommendation lies in data suggesting that even after closuring, those patients have a high risk of recurrence, requiring antithrombotic therapy anyway [9-10].

## Conclusions

While significant progress has been made with the International CAPS Registry, there are still several gaps in our knowledge about this rare and frequently fatal condition. While PFO seems to be even more preventable in this group of patients compared with the general population, the contribution of concomitant PFO to APS patients' thrombotic risk is still unknown. With this complex and challenging case report and review, we want to be aware of the risk of misdiagnosing CAPS when a patient with APS presents with multiple arterial and venous events in the absence of microvascular thrombotic manifestations, while this can be justified with the concomitant presence of a PFO.
